# Simultaneous pyometra and viable puppies’ gestation in a bitch

**Published:** 2014-08-24

**Authors:** A. Risso, F.J. Pellegrino, Y. Corrada

**Affiliations:** 1 *Hospital Escuela, Facultad de Ciencias Veterinarias (FCV), Universidad Nacional de La Plata (UNLP), 60 y 118 S/N, La Plata, Buenos Aires, Argentina*; 2 *Consejo Nacional de Investigaciones Científicas y Técnicas (CONICET), Buenos Aires, Argentina*

**Keywords:** Bitch, Gestation, Pyometra, Viable puppies

## Abstract

Here we describe a case of pyometra coexisting with gestation in a 4.5 year-old miniature short-haired Dachshund. The dog exhibited depression, vaginal discharge, polydipsia and dehydration. Ultrasound examination revealed the presence of low to moderate anechoic fluid collection in the left uterine horn. Blood analysis revealed mild neutrophilia with a left shift. Based on these findings a presumptive diagnosis of pyometra was made and the bitch was treated using amoxicillin-clavulanate with dopaminergic agonist (cabergoline). A second ultrasound scan revealed the presence of two gestational vesicles in the right uterine horn that were successfully carried to term. Unusually, while pyometra persisted in the left uterine horn, two viable puppies were delivered by caesarean section from the right uterine horn.

## Introduction

Cystic endometrial hyperplasia-pyometra complex is the accumulation of purulent material within the uterine lumen of the bitch that occurs during or after a period of progesterone dominance (Pretzer, 2008). It tends to affect middle and old aged female dogs, but can also affect young females receiving hormonal treatments (Whitehead, 2008). In canines, progesterone plays a key role in the maintenance of gestation because of its effects on endometrial glands, myometrial contractions and uterine immunity (Feldman and Nelson, 1996).

Therefore, the environment necessary to carry out a normal gestation may become an optimal medium for undesirable bacterial growth. Orozco *et al*. (2005) reported the simultaneous presence of pregnancy and pyometra in a bitch that delivered two stillborn puppies. This report describes a successful delivery of two viable pups in a bitch that was treated for pyometra during pregnancy.

### Case details

A 4.5 year-old miniature short-haired Dachshund bitch (5.4 kg) was presented to the Veterinary Hospital of the Faculty of Veterinary Medicine, National University of La Plata with symptoms of depression, vaginal discharge and polydipsia, 22 days after mating (30 days after her sixth and final oestrus).

The bitch had a history of a previous caesarean section where two viable puppies (out of five gestational vesicles) were born after her third oestrous cycle. The dog developed an open-cervix pyometra in the luteal phase of the fifth oestrous cycle that was successfully treated with a combination of amoxicillin-clavulanate, but a moderate endometrial hyperplasia persisted as revealed by an ultrasound scan.

Clinical evaluation confirmed a purulent vulvar discharge, accompanied by mild dehydration, and a rectal temperature of 39.5°C. Blood samples were obtained by peripheral venipuncture and submitted for laboratory analysis, and finally, a uterine ultrasound scan was performed to diagnose possible recurrence of pyometra as proposed by Bigliardi *et al*. (2004). Blood concentrations of urea, creatinine, albumin, total protein, glucose, and haemoglobin, as well as haematocrit, and red and total white blood cell counts were within their respective reference ranges. The only relevant finding was a mild neutrophilia with a left shift (700 cells/µl).

Ultrasound examination with 8-MHz linear transducer (Toshiba Core Vision Pro, Japan) revealed the presence of a small to moderate collection of anechoic fluid in the left uterine horn. Pyometra was suspected and the dog received 5 µg/kg PO q 24 hours cabergoline (Relay®, Holliday) for 7 days, together with a combination of 12.5 mg/kg bid PO amoxicillin-clavulanate (Clavamox®, Pfizer), which was given for 10 days (Corrada *et al.*, 2006). In addition, supportive hydration care was provided for 10 days as follows: fluid therapy (60 ml/kg +% dehydration x body weight/100) to correct dehydration, for the first 48 hours, followed by oral rehydration salts for another 8 days.

A second ultrasound examination five days later confirmed the presence of two gestational vesicles in the right uterine horn and the collection of anechoic fluid in the left uterine horn. Favourable clinical response to treatment was confirmed by the restoration of normal body temperature and recovery of normal attitude that were maintained throughout the gestation period. Clinical re-examinations and ultrasound scans were performed weekly to detect any possible resorption of pregnancy vesicles. Antibiotic therapy was prolonged for a further 10 days.

The ultrasound examinations revealed normal growth of the two pregnancy vesicles. Foetal viability was tested by ultrasound scan and by daily foetal doppler during the last 10 days of gestation. A radiological study, performed a week prior to the caesarean section, confirmed the presence of two foetuses in the right uterine horn. Caesarean section followed by ovariohysterectomy was performed once foetal maturity was confirmed by ultrasound examination, and when the heart rate fell below 170 beats per minute. The surgical procedure was carried out as previously described (Gilson, 2003).

**Fig. 1:**
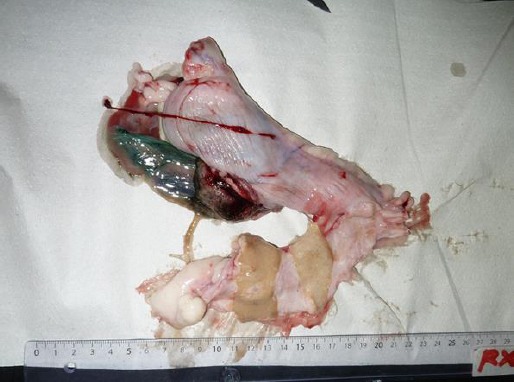
Picture showing the uterus of a pregnant miniature short-haired Dachshund bitch with pyometra. Shown is the placenta in the right uterine horn (top) and the purulent material in the left horn (bottom). The ruler’s scale is in centimetres.

After premedication with 0.2 mg/kg IV diazepam (Diazepam®, Lamar), general anaesthesia was induced with 2.0 mg/kg IV propofol (Gobbifol®, Gobbi Novag SA), and maintained with isoflurane (Isoflurane®, Baxter International) and oxygen at 100%. A male and a female pup were delivered live from the right uterine horn, while abundant collection of fluid was noted in the left uterine horn. This was confirmed as purulent material by opening the left uterine horn (Figure 1).

## Discussion

There is little information about simultaneous occurrence of pyometra and pregnancy in bitches (Orozco *et al.*, 2005).

This present report confirms that it is possible for a bitch to have both pyometra in one uterine horn and a successful pregnancy in the other with proper medical treatment. On presentation, the dog had a prior history of pyometra, and therefore its recurrence was feasible as reported by Arnold (2006). The infection could have occurred at the time of mating as a result of a pre-existing vaginal infection or a pre-existing endometrial hyperplasia, or both.

It is generally accepted that hyperplasia of endometrial glands and reduced myometrial contractions during diestrus (progesterone effect) increase the risk of pyometra (Feldman, 2000; Orozco *et al.*, 2005). Therefore embryo implantation could have occurred in one horn, whereas in the other pyometra could have developed as a consequence of the physiological changes induced by progesterone in the endometrial structure.

It is reported that dopaminergic agonists act indirectly on the corpus luteum by withdrawing the main luteotropic hormone during the second half of the luteal phase (Onclin *et al.*, 1993). The lack of effect of cabergoline in this case could be due to the probable multifactorial regulation of corpus luteum activity during the first half of gestation (Onclin *et al.*, 1993; Kowaleski, 2012).

Therefore, corpora lutea would not be affected by the luteotropic hormone withdrawal induced by the treatment with cabergoline.

## Conclusion

A novel presentation of canine pregnancy with coexisting pyometra is reported here. Although medical treatment failed to fully resolve the pyometra in the left uterine horn, it may have prevented further progression, helping to maintain the viability of the gestational vesicles to term in the contralateral uterine horn, allowing the birth of two viable puppies.

## References

[ref1] Arnold S (2006). Canine pyometra: new approaches to an old disease.

[ref2] Bigliardi E, Parmigiani E, Cavirani S, Luppi A, Bonati L, Corradi A (2004). Ultrasonography and cystic hyperplasia-pyometra complex in the bitch. Reprod. Domest. Anim.

[ref3] Corrada Y, Arias D, Rodríguez R, Tórtora M, Gobello C (2006). Combination dopamine agonist and prostaglandin agonist treatment of cystic endometrial hyperplasia-pyometra complex in the bitch. Theriogenology.

[ref4] Feldman E, Ettinger S, Feldman E (2000). The cystic endometrial hyperplasia pyometra complex and infertility in female dogs. Textbook of Veterinary Internal Medicine disease of the dog and cat.

[ref5] Feldman E.C, Nelson R (1996). Canine and Feline Endocrinology and Reproduction.

[ref6] Gilson S.D, Slatter D.P.A (2003). Cesarean section. Textbook of small animal surgery.

[ref7] Kowaleski M (2012). Endocrine and molecular control of luteal and placental function in dogs.

[ref8] Onclin K, Silva L.D, Donnay I, Verstegen J.P (1993). Luteotrophic action of prolactin in dogs and the effects of a dopamine agonist, cabergoline. J. Reprod. Fertil. Suppl.

[ref9] Orozco P, Quiroz H, Gomez G, Villegas T (2005). Piómetra y gestación simultáneos en una perra: reporte de un caso. R. C. C. P.

[ref10] Pretzer S.D (2008). Clinical presentation of canine pyometra and mucometra: A review. Theriogenology.

[ref11] Whitehead M.L (2008). Risk of pyometra in bitches treated for mismating with low doses of oestradiol benzoate. Vet. Rec.

